# Author Correction: Highly confined epsilon-near-zero and surface phonon polaritons in SrTiO_3_ membranes

**DOI:** 10.1038/s41467-024-52983-2

**Published:** 2024-10-02

**Authors:** Ruijuan Xu, Iris Crassee, Hans A. Bechtel, Yixi Zhou, Adrien Bercher, Lukas Korosec, Carl Willem Rischau, Jérémie Teyssier, Kevin J. Crust, Yonghun Lee, Stephanie N. Gilbert Corder, Jiarui Li, Jennifer A. Dionne, Harold Y. Hwang, Alexey B. Kuzmenko, Yin Liu

**Affiliations:** 1 Department of Materials Science and Engineering, North Carolina State University, Raleigh, NC 27606 USA; 2 Department of Quantum Matter Physics, University of Geneva, 1211 Geneva, Switzerland; 3 Advanced Light Source Division, Lawrence Berkeley National Laboratory, Berkeley, CA 94720 USA; 4 Beijing Key Laboratory of Nano-Photonics and Nano-Structure (NPNS), Department of Physics, Capital Normal University, Beijing, China; 5 Department of Physics, Stanford University, Stanford, CA 94305 USA; 6 Stanford Institute for Materials and Energy Sciences, SLAC National Accelerator Laboratory, Menlo Park, CA 94025 USA; 7 Department of Applied Physics, Stanford University, Stanford, CA 94305 USA; 8 Department of Materials Science and Engineering, Stanford University, Stanford, CA 94305 USA

**Keywords:** Optical materials and structures, Nanoscale materials, Nanophotonics and plasmonics, Two-dimensional materials

Correction to: *Nature Communications* 10.1038/s41467-024-47917-x, published online 04 June 2024

In this article the grant number #200020_201096 relating to the Swiss National Science Foundation was omitted. The original article has been corrected.

