# Supplementary material for: Highly confined epsilon-near-zero and surface phonon polaritons in SrTiO3 membranes
Source: Nat Commun. 2024 Jun 4;15:4743. doi: 10.1038/s41467-024-47917-x (PMC11150425; doi:10.1038/s41467-024-47917-x)
Supplement: Supplementary file 1 — Supplementary information [file 41467_2024_47917_MOESM1_ESM.pdf]

Supplementary Information: Highly confined epsilon-near-zero and surface phonon polaritons in $SrTiO_3$ membranes

Ruijuan Xu[1§], Iris Crassee[2§], Hans A. Bechtel[3], Yixi Zhou[2,4], Adrien Bercher[2], Lukas Korosec[2], Carl Willem Rischau[2], Jérémie Teyssier[2], Kevin J. Crust[5,6], Yonghun Lee[6,7], Stephanie N. Gilbert Corder[3], Jiarui Li[6,7], Jennifer A. Dionne[8], Harold Y. Hwang[6,7], Alexey B. Kuzmenko[2]*, and Yin Liu[1]*

[1]Department of Materials Science and Engineering, North Carolina State University, Raleigh, NC 27606, USA

[2]Department of Quantum Matter Physics, University of Geneva, 1211 Geneva, Switzerland

[3]Advanced Light Source Division, Lawrence Berkeley National Laboratory, Berkeley, CA 94720, USA

[4]Beijing Key Laboratory of Nano-Photonics and Nano- Structure (NPNS), Department of Physics, Capital Normal University, 100048 Beijing, China

[5]Department of Physics, Stanford University, Stanford, CA, 94305, USA

[6]Stanford Institute for Materials and Energy Sciences, SLAC National Accelerator Laboratory, Menlo Park, CA 94025, USA

[7]Department of Applied Physics, Stanford University, Stanford, CA 94305, USA

[8]Department of Materials Science and Engineering, Stanford University, Stanford, CA 94305, USA

*Emails: Alexey.Kuzmenko@unige.ch and yliu292@ncsu.edu

§R.X. and I.C. contributed equally to this paper

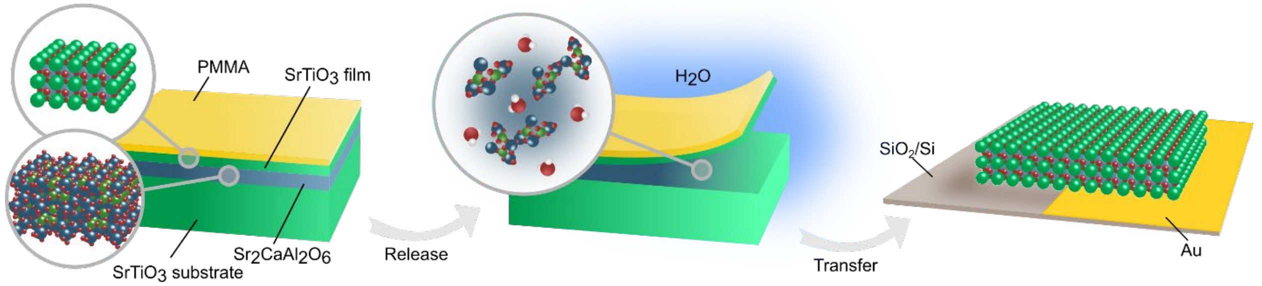

Supplementary Figure 1. Schematic illustration of the lift-off and transfer process for preparing $SrTiO_3$ membranes on SiO_2/Si substrates. By dissolving the sacrificial layer $Sr_2CaAl_2O_6$ from the as-grown heterostructure, $SrTiO_3$ films can be released and transferred with a support layer of PMMA onto a SiO_2/Si substrate which is half coated by 50 nm gold. The PMMA support layer was then removed by dissolving in acetone at 60 °C and then washing in isopropanol, leaving just the $SrTiO_3$ membrane on the substrate.

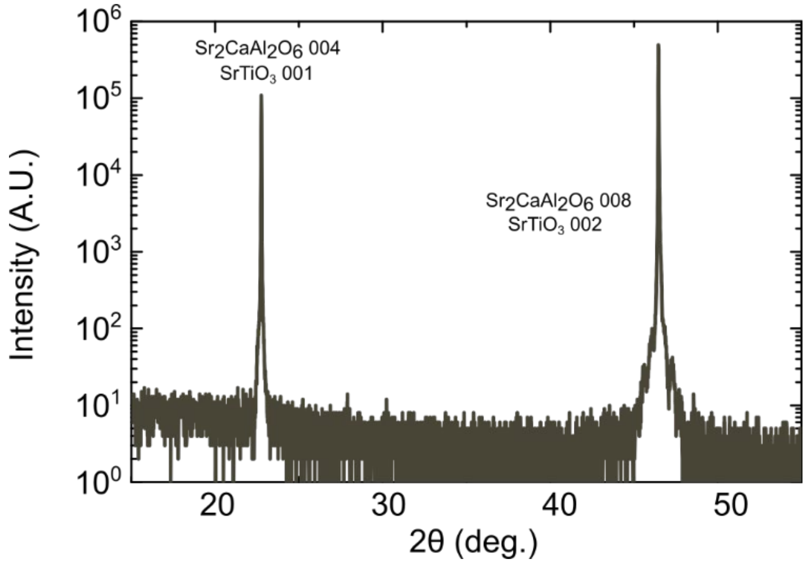

Supplementary Figure 2. θ - 2θ x-ray diffraction scans showing a single diffraction peak with all layer peaks overlapping together due to the small lattice mismatch between the different film layers, indicating that the as grown films are epitaxial, single-phase.

Supplementary note 1. Far-field infrared reflectivity and dielectric function of bulk $SrTiO_3$

The dielectric function of bulk $SrTiO_3$, $\varepsilon_{STO}(\omega)$, was determined by measuring the reflectivity $R_{STO}(\omega)$ at near-normal angle of incidence (Supplementary Fig. 3) and fitting it with the dielectric function parameterized with the factorized formula[1]:

$$\varepsilon_{STO}(\omega) = \varepsilon_\infty \times \prod_{i=1}^{N} \frac{\omega_{LO,i}^2 - \omega^2 - i\gamma_{LO,i}\omega}{\omega_{TO,i}^2 - \omega^2 - i\gamma_{TO,i}\omega}$$

and the Fresnel equation:

$$R_{STO}(\omega) = \left|\frac{1-\sqrt{\varepsilon_{STO}(\omega)}}{1+\sqrt{\varepsilon_{STO}(\omega)}}\right|^2.$$

The best fit (solid line in Supplementary Fig. 3) to our data is obtained with $\varepsilon_\infty = 5.63 \pm 0.04$ and three oscillators as shown in the Supplementary Table 1. These parameters are close to the ones found in the literature[1,2].

Supplementary Table 1

i	$\omega_{TO,i}$ (cm^{-1})	$\gamma_{TO,i}$ (cm^{-1})	$\omega_{LO,i}$ (cm^{-1})	$\gamma_{LO,i}$ (cm^{-1})
1	94.6±1.3	11.8±1.9	172.0±0.3	2.6±0.6
2	175.0±0.6	4.5±1.0	475.0±0.3	5.0±0.5
3	546.1±0.8	17.6±1.1	798.4±1.7	27.7±2.3

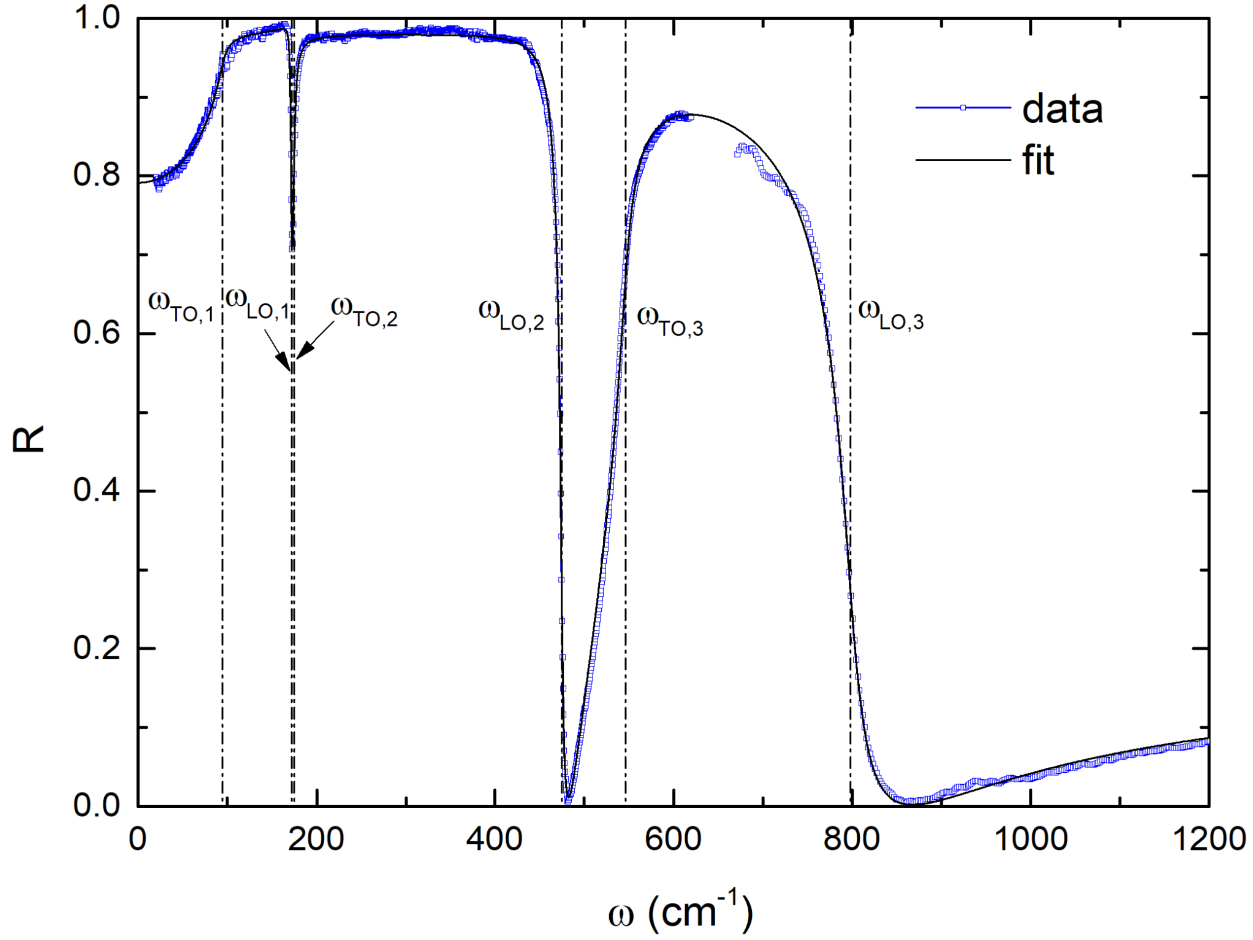

Supplementary Figure 3. Far-field FTIR reflectivity spectra of a bulk $SrTiO_3$ crystal at 300 K. The solid line is a model fit obtained as described in the text. The vertical lines represent the TO and LO frequencies from Supplementary Table 1.

The penetration depth $\delta_{STO}(\omega) = \frac{c}{\omega\, Im[\sqrt{\varepsilon_{STO}(\omega)}]}$ is shown in Supplementary Fig.4 (blue line). One can see that it is much longer than the thickness of the membrane (red line).

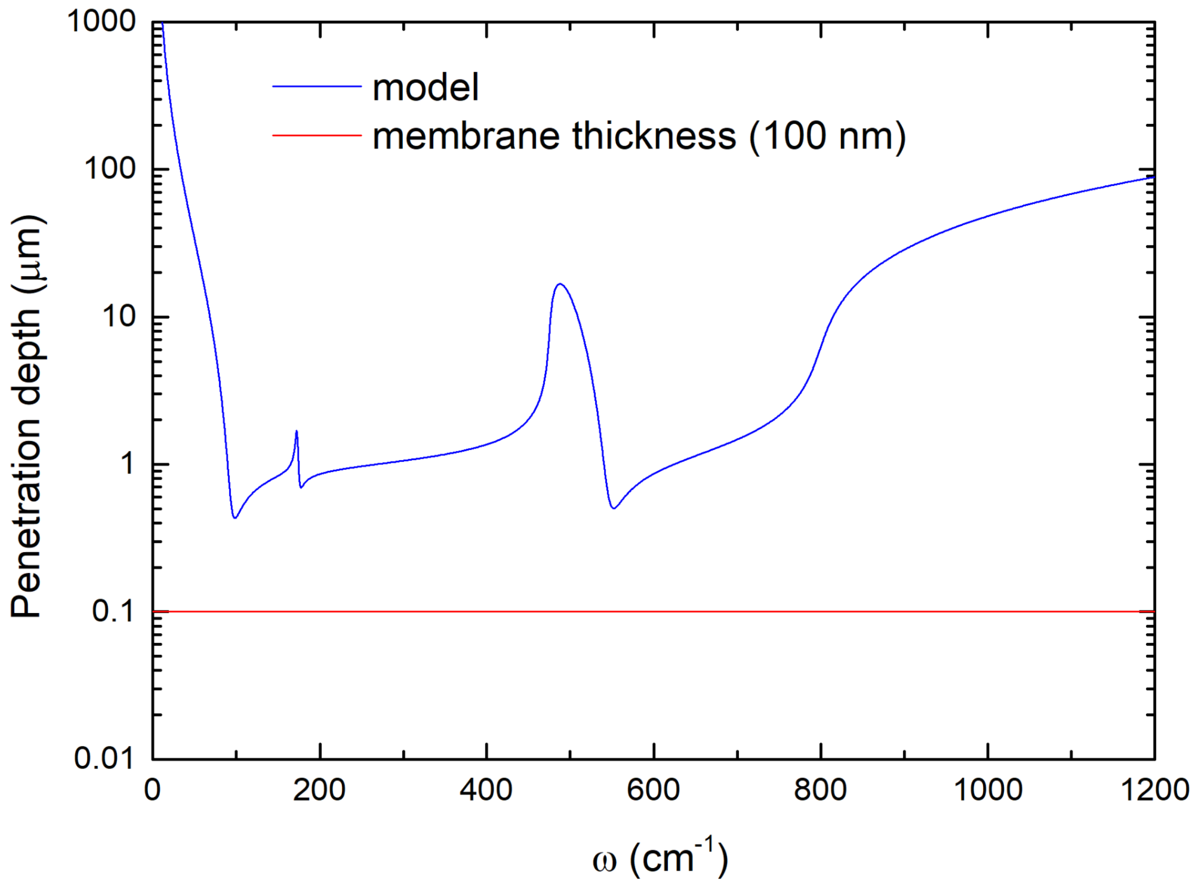

Supplementary Figure 4. Penetration depth of $SrTiO_3$ (blue line) as compared to the membrane thickness (red line).

Supplementary note 2. Dependence of the Berreman mode intensity on the angle of incidence

In Supplementary Fig.5, we compare the far-field reflectivity spectra of the $SrTiO_3$ on gold obtained with two different reflective objectives having the numerical apertures of 0.4 (brown symbols) and 0.5 (blue symbols). The second objective has a higher value of the average angle of incidence and therefore the z-axis component of the incident electromagnetic field for the p-polarized light is expected to be larger. Accordingly, the Berreman mode intensity is clearly higher for the second objective. The solid and dashed lines are model calculations using the angle of incidence of 13 and 18 degrees. For these values, a good match is obtained for the first and the second objective respectively. Given than the light is unpolarized and therefore contains about 50 percent of the s-polarization, for which the z-axis component of the field is zero and also taking

into account that the actual distribution of the angles of incidence depends not only on the numerical aperture but also on the actual alignment of the infrared beam in the microscope, we limit ourselves with stating that these angle values qualitatively agree with the specifications of the objectives.

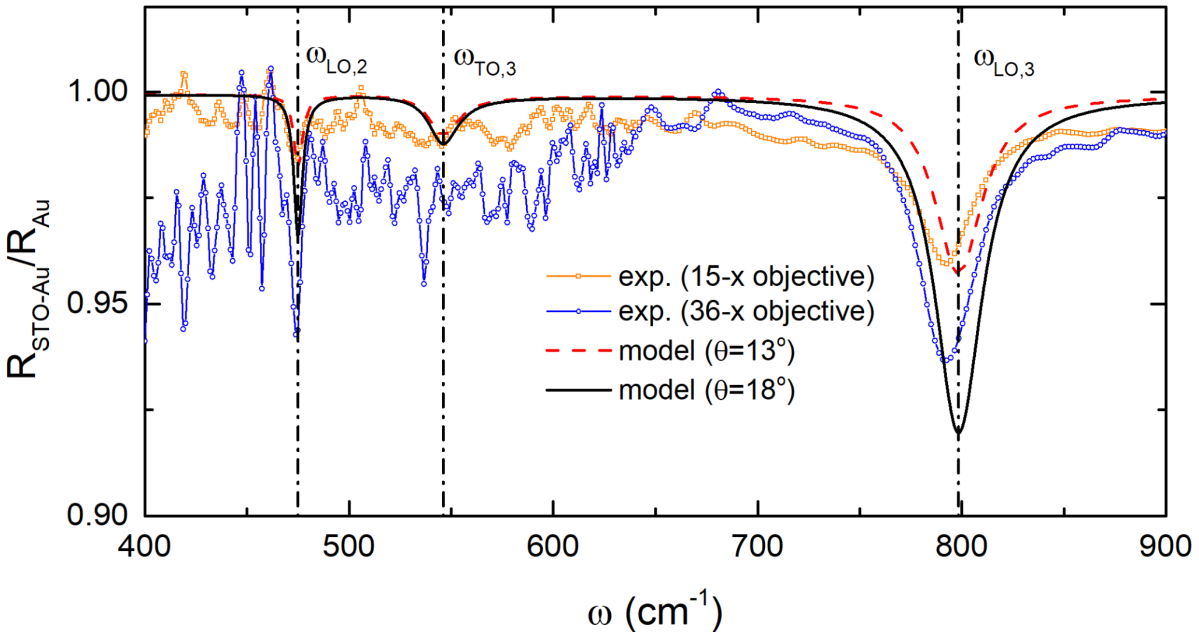

Supplementary Figure 5. Far-field infrared reflectivity of a 100 nm thick $SrTiO_3$ membrane on gold. Orange and blue symbols - measurements with two reflective Cassegrain objectives (15- and 36-fold amplification respectively). Dashed and solid lines - calculated spectra using the dielectric function of bulk $SrTiO_3$ for the two angles of incidence ($\theta = 13^o$ and 18° correspondingly).

Supplementary note 3. Far-field FTIR characterization of the bare SiO_2/Si substrate

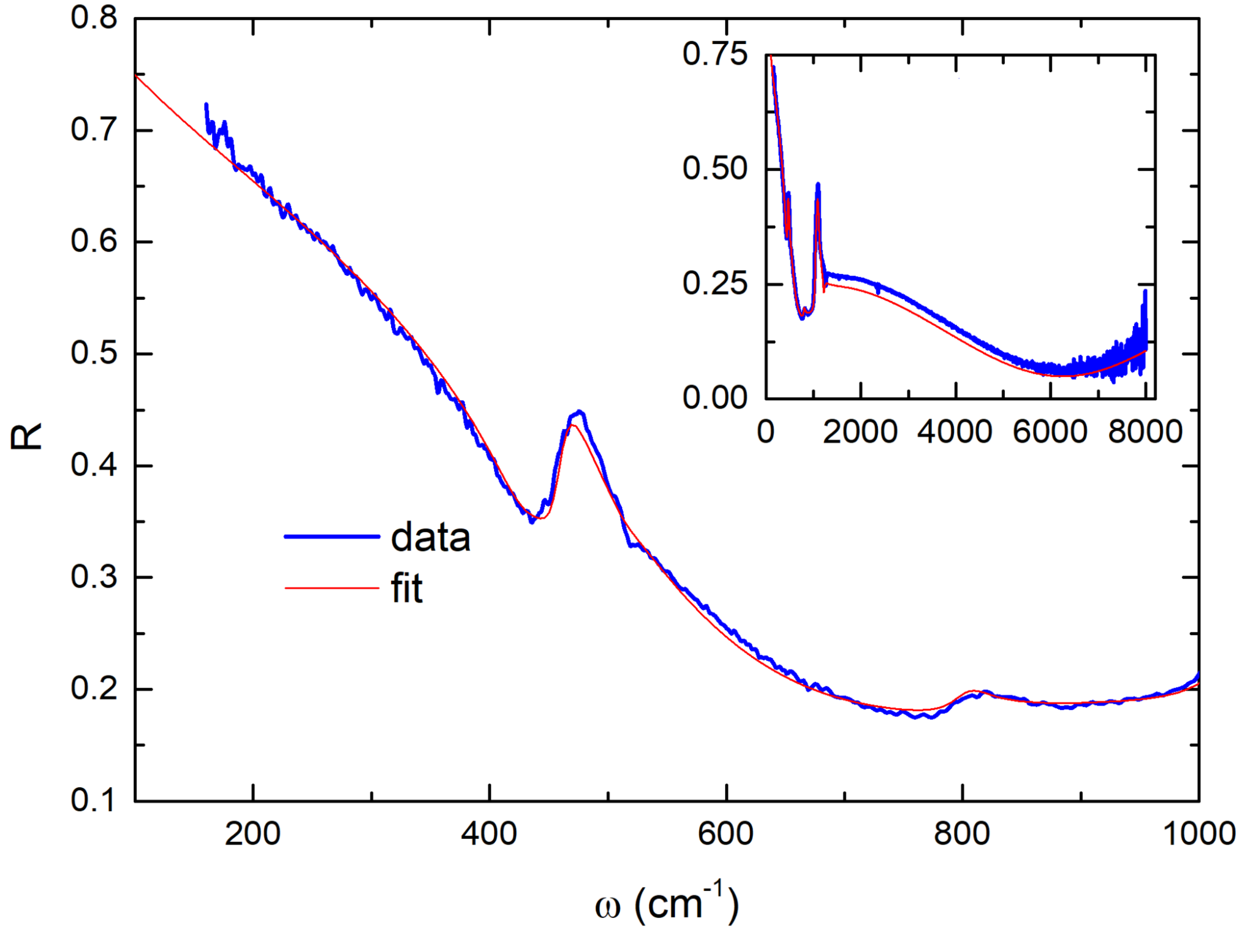

Supplementary Figure 6. Far-field FTIR reflectivity of bare SiO_2/Si substrate at room temperature in the spectral range relevant for other optical measurements in the paper. The model curves are obtained by a least square fitting where the Drude-Lorentz parameters of doped Si were adjusted. The inset shows the same data and model in a broad range, emphasizing the Fabry-Pérot minimum due to the interference in the SiO_2 layer.

The reflectivity spectrum of SiO_2/Si is shown with blue symbols in Supplementary Fig.6 (inset shows an extended range of frequencies). The measurement is done on the same sample as the one used in the paper, and the Au-covered area was used for the normalization. The spectrum is marked with three SiO_2 phonon structures (at about 450, 800 and 1100 cm^{-1}). The rise of reflectivity below 600 cm^{-1} is due to the Drude response of doped charge carriers in Si.

In order to model these data, one has to know the dielectric function of SiO_2 and Si as well as the thickness of the oxide layer. The dielectric function of SiO_2 is well known. In particular, we used the dielectric function of thermal SiO_2 deposited on Si obtained in Ref [3] using spectroscopic ellipsometry. We modeled it by a sum of Lorentz oscillators:

$$\varepsilon_{SiO_2}(\omega) = \varepsilon_\infty + \sum_{i=1}^{N} \frac{\omega_{p,i}^2}{\omega_{0,i}^2 - \omega^2 - i\gamma_i\omega}.$$

The parameters used are the following: $\varepsilon_\infty = 2.19 \pm$ *0.05* and the rest of them are collected in this table:

Supplementary Table 2

i	$\omega_{0,i}$ (cm^{-1})	$\omega_{p,i}$ (cm^{-1})	γ_i (cm^{-1})
1	456.2±0.2	418.2±3.2	37.0±0.7
2	803.0±4.6	153.8±21.0	32.9±12.9
3	1075±0.5	829.3±8.4	49.4±4.5

Afterwards, the thickness of SiO_2 (275 nm) was deduced from the position of the reflectance interference minimum at about 6000 cm^{-1} (see the inset in Supplementary Fig. 6).

Finally, we fitted the experimental data using the Drude model for doped silicon:

$$\varepsilon_{Si}(\omega) = \varepsilon_\infty - \frac{\omega_p^2}{\omega(\omega + i\gamma\omega)}.$$

The parameters, which result from the fit, are the following: the high-frequency dielectric function $\varepsilon_\infty = 11.6 \pm 0.02$, the plasma frequency $\omega_p = 2138.6$ cm^{-1} and the scattering rate $\gamma = 470.5$ cm^{-1}. The model curve (solid line in Supplementary Fig.6), reproduces the data very well below 1000 cm^{-1}, which is the range important for this paper. The model curve is lower by a few percent than the experimental data at higher frequencies, which is possibly due to the deviation of the reflectivity of the reference gold from unity.

Supplementary note 4. Some phonon frequencies of $SrTiO_3$ obtained by fitting the far-field reflectivity on the membrane

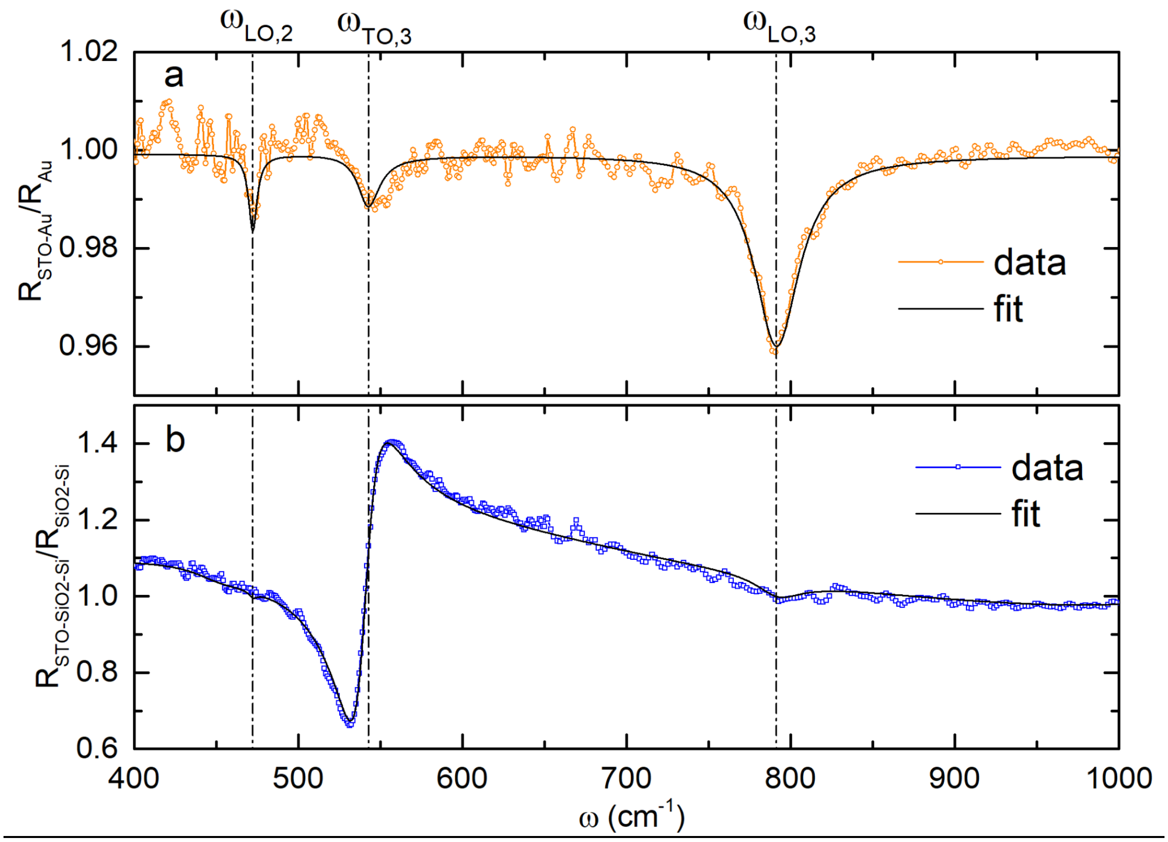

Supplementary Figure 7. Model curves obtained by least-square fitting of the far-field reflectivity measured of the $SrTiO_3$ membrane on SiO_2/Si (divided by bare SiO_2/Si) (**a**) and on Au (divided by Au) (**b**). The vertical lines correspond to the refined values of the phonon frequencies, which are slightly different from the one obtained on a bulk $SrTiO_3$ sample (Supplementary Fig.3 and Supplementary Table 1)

Some phonon frequencies in STO can be obtained by direct least-square fitting of the far-field reflectivity curves (Supplementary Fig. 7). These values are presented in Supplementary Table 3.

Supplementary Table 3

i	$\omega_{TO,i}$ (cm^{-1})	$\gamma_{TO,i}$ (cm^{-1})	$\omega_{LO,i}$ (cm^{-1})	$\gamma_{LO,i}$ (cm^{-1})
2	-	-	472.2±0.8	6.3±1.7
3	542.7±0.4	16.2±0.7	791.3±1.1	33.1±2.3

Supplementary note 5. Comparison between the near-field spectra of $SrTiO_3/SiO_2/Si$ and bare SiO_2/Si

In supplementary Fig. 8a and 8c, we compare the near-field amplitude spectrum on the membrane lying on top of SiO_2/Si with the spectrum on bare substrate. This shows us that the phonon modes from silicon oxide are present in both cases.

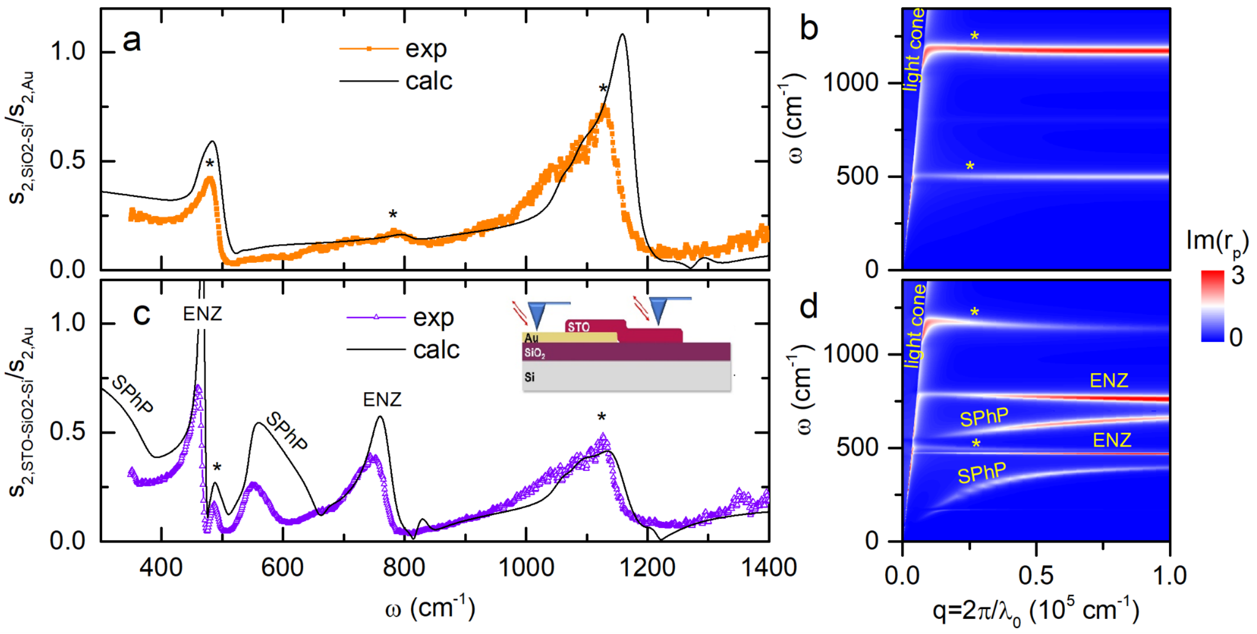

Supplementary Figure 8. a and **c,** Near-field nano-FTIR spectra of the 2nd harmonics of the SINS amplitude, s_2, normalized by the signal on gold, $s_{2,Au}$, obtained from bare SiO_2/Si (a) and nanomembrane of $SrTiO_3$ on SiO_2/Si (c). The symbols are measurements and solid lines are finite-dipole model simulations (using the dielectric function of bulk $SrTiO_3$. **b** and **d,** the corresponding dispersion maps.

Supplementary note 6. Complex-valued analysis of near-field line profiles of the $SrTiO_3$ membrane.

The complex-valued near-field signal, denoted as $s_2(x)e^{i\phi_2(x)}$ and constructed from the experimental data , exhibits a well-fitting behavior described by $A\frac{e^{iqx}}{x}+B$ (as indicated by the red lines in Fig. 4e, f and Supplementary Fig. 9). Here, A and B represent complex fitting parameters, and q is the complex wavevector ($q = q_1 + iq_2$). This outcome implies that the detected polariton signals are primarily influenced by the polaritons initiated at the edge and subsequently scattered by the tip.

Furthermore, FDTD simulation is conducted to elucidate the field distribution of surface phonon polaritons (SPhPs) launched from the edge (refer to Supplementary Fig. 10). These findings can be rationalized by considering the noticeable damping of the SPhPs. As polaritons launched from the edge cover only half the distance, they experience comparatively less damping than both tip-launched and edge-reflected polaritons, which travel a distance of $2x$.

To enhance clarity in illustrating the propagating modes, we subtract the offset parameter B from the complex-valued data, representing it as $\sigma^* = s_2^* e^{i\phi_2^*}$. The decaying amplitude (s_2^*) and a linearly increasing phase (ϕ_2^*) confirm the propagating nature of the modes with damping (Supplementary Fig. 9b and 9c).

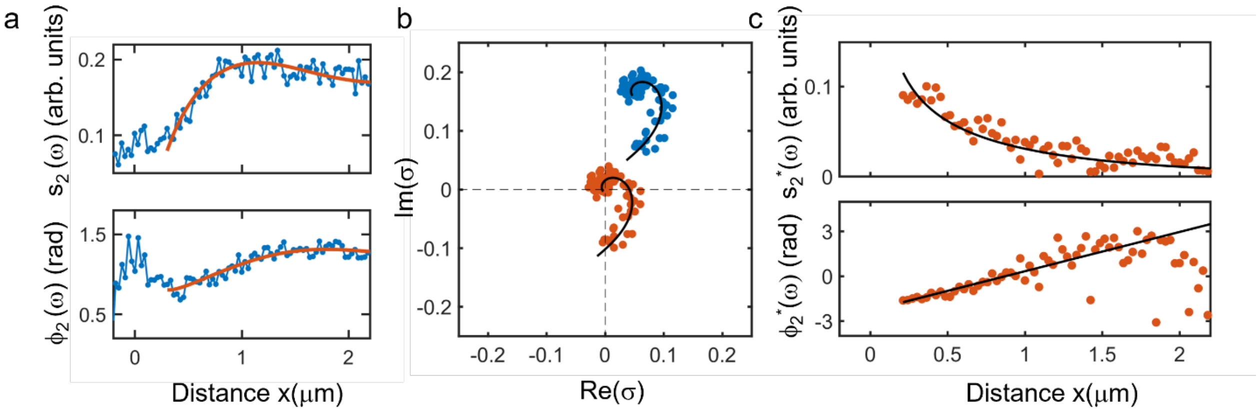

Supplementary Figure 9. a Near-field amplitude (upper) and phase (lower) line profiles of the $SrTiO_3$ film simultaneously at 574 cm^{-1}. Red solid lines show the fitting of the experimental data from a complex-valued function $E_{PhP} = A\frac{e^{iqx}}{x} + B$. **b** Representation of the near-field line profiles in the complex plane. Blue dots show the data constructed from the measured amplitude and phase. Red dots show the data after the subtraction of complex-valued signal offset B at large distance x. Black solid lines show the fittings. **c** Amplitude (upper) and phase (lower) line profiles obtained from the data shown in **b** after subtraction of the complex-valued signal offset B.

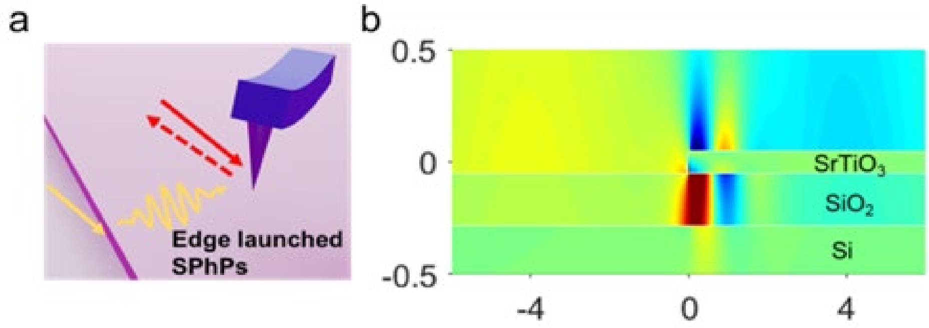

Supplementary Figure 10. a,b Schematic (**a**) and simulated E_z field-distribution (**b**) showing the SPhPs launched by the edge of the membrane.

Supplementary note 7. Real-space SINS nanoimaging of gold-supported $SrTiO_3$ membranes

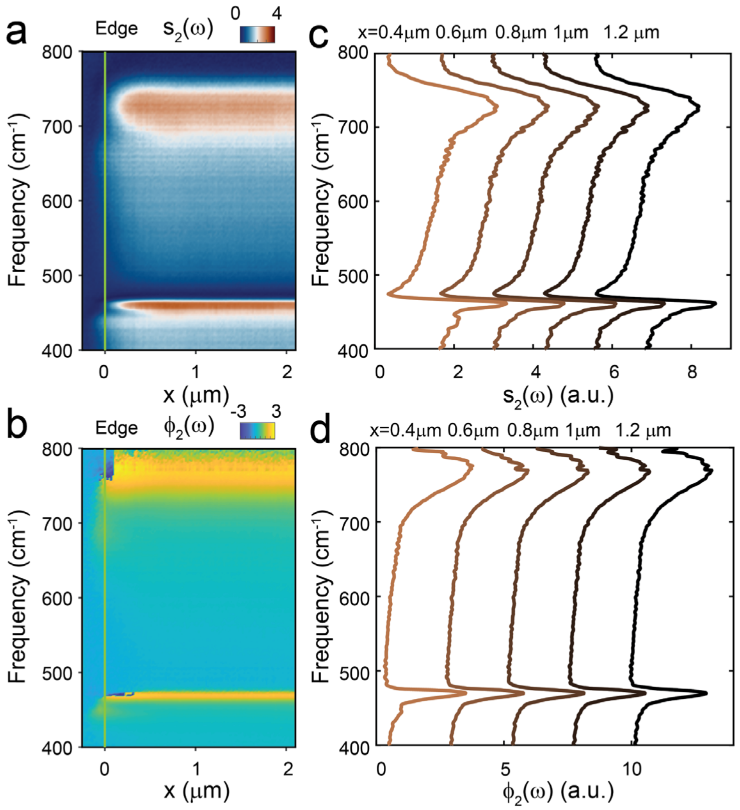

Supplementary Figure 11. a,b Experimental near-field amplitude (**a**) and phase (**b**) spectra obtained by a line scan perpendicular to the edge of the membrane on gold substrates. **c,d** Near-field amplitude (**d)** and phase (**d**) spectra obtained at locations on the gold supported membrane with different distances to the edge. The edge located at x=0 is denoted by the green line.

References

1. Kamarás, K. *et al.* The low-temperature infrared optical functions of $SrTiO_3$ determined by reflectance spectroscopy and spectroscopic ellipsometry. *J. Appl. Phys.* **78**, 1235–1240 (1995).
2. Servoin, J. L., Luspin, Y. & Gervais, F. Infrared dispersion in $SrTiO_3$ at high temperature. *Phys. Rev. B Condens. Matter* **22**, 5501–5506 (1980).
3. Ji, Y.-Q. *et al.* Optical constants of SiO_2 films deposited on Si substrates. *Chin. Physics Lett.* **31**, 046401 (2014).
